# *In vitro* anthelmintic activity of *Phyllanthus niruri* Linn., *Andrographis paniculata*, *Curcuma xanthorrhiza* Roxb., and *Curcuma aeruginosa* Roxb. ethanol extracts on the motility and cuticle damage of *Ascaridia galli*

**DOI:** 10.14202/vetworld.2024.2488-2496

**Published:** 2024-11-07

**Authors:** Risa Tiuria, Lina Noviyanti Sutardi, Arifin Budiman Nugraha, Mawar Subangkit

**Affiliations:** 1 Division of Parasitology and Medical Entomology, School of Veterinary Medicine and Biomedical Sciences, IPB University, Bogor, Indonesia; 2 Division of Pharmacy, School of Veterinary Medicine and Biomedical Sciences, IPB University, Bogor, Indonesia; 3 Division of Pathology, School of Veterinary Medicine and Biomedical Sciences, IPB University, Bogor, Indonesia

**Keywords:** *Ascaridia galli*, cuticle, *in vitro* motility, plant extract

## Abstract

**Background and Aim:**

*Ascaridia galli*, a nematode that frequently infects the digestive tract of chickens, is a significant concern for poultry health. In response, the use of medicinal plant-derived anthelmintics was proposed as a potential solution. This study observed the *in vitro* effectiveness of a single, graded dose of the ethanol extract of *Andrographis paniculata*, *Phyllanthus niruri* L., *Curcuma xanthorrhiza* Roxb., *and Curcuma aeruginosa* Roxb. on the movement activity of adult *A. galli* every hour for 6 h, followed by an analysis of worm cuticle damage in *A. galli*.

**Materials and Methods:**

A randomized block design was used. Adult *A. galli* were collected from the intestinal lumen of fresh free-range chickens. Each petri dish contained two *A. galli* for each treatment with three replications. Each plant extract (*A. paniculata, P. niruri* L., *C. xanthorrhiza* Roxb., and *C. aeruginosa* Roxb.) was evaluated with three distinct doses, which were 250 μg/mL, 500 μg/mL, and 1000 μg/mL; 0.9% sodium chloride solution was used as a negative control, and 500 μg/mL Albendazole solution was used as a positive control. The active compound content of *A. paniculata*, *P. niruri* L., *C. xanthorrhiza* Roxb., and *C. aeruginosa* Roxb. extracts were analyzed using ultra-performance liquid chromatography-mass spectrometry. The movement activity of *A*. *galli* was determined by the percentage score value from the 1^st^ to the 6^th^ h in each treatment group, followed by analysis of damage to the *A. galli* cuticle layer using a nano-microscope and histopathological images.

**Results:**

Analysis of variance demonstrated that at doses of 250 μg/mL and 500 μg/mL, the ethanol extracts of *A. paniculata*, *P. niruri* L., *C. xanthorrhiza* Roxb., and *C. aeruginosa* Roxb. did not have a significant effect on the effectiveness of *A. galli*’s *motility* (>0.005). However, at a dose of 1000 μg/mL, the ethanol extract of *A. paniculata*, *P. niruri* L., *C. xanthorrhiza* Roxb., and *C. aeruginosa* Roxb. reduced the motility of *A. galli*. Importantly, the motility of *A. galli* in the dose of 1000 μg/mL *A. paniculata* and *P. niruri* L. extract groups was very weak and significantly different (p < 0.001) compared to the negative control group. The content of the active compound Andrographolide in the ethanol extract of *A. paniculata* and the active compound 5-Methoxybenzimidazole in the extract of *P. niruri* L. are strongly suspected to play an important role in damaging and shedding the cuticle layer of *A*. *galli*.

**Conclusion:**

All herbal extracts have anthelmintic activity at a concentration of 1000 μg/mL. Extracts of *A. paniculata*, *P. niruri* L., *C. xanthorrhiza* Roxb., and *C. aeruginosa* Roxb. have activities that can damage and dissolve the cuticle layer of *A*. *galli*, resulting in the weakening of the motility of *A. galli*.

## Introduction

Chicken is a poultry commodity and an important source of animal protein. Several factors, such as poultry diseases, affect poultry farming. One chronic poultry disease with an economic impact is infection by a parasitic worm. *Ascaridia galli* is a nematode that has become the most common helminth parasite in poultry. *A. galli* is a significant parasitic nematode that affects poultry, particularly in Indonesia and globally. *A. galli* infection in chickens has various harmful effects on the health and productivity of animals. The results of malnutrition, emaciation, malabsorption, and anemia. It also suppresses the immune system and renders chickens more vulnerable to other infections at the same time. The effects of the parasite on chicken health are further complicated by its capacity to serve as a vector for various infections [1–3].

The routine commercial use of anthelmintics can lead to several problems, including the development of worm resistance, environmental pollution, and accumulation of drug residues in tissues. Among the alternatives to anthelmintics, natural products are more environmentally friendly, consumer-friendly, and host-friendly because of their lower or no toxic effects. Resistance of anthelmintics to *A. galli* is a major concern in poultry farming. The emergence of drug resistance has hampered the use of commercial synthetic anthelmintics and is frequently attributed to incomplete treatment with prescribed doses and withdrawal times. This resistance has necessitated the development of more sustainable approaches for parasitic infection control in poultry [4, 5]. Using medicinal herbs as traditional medicine is one of the potential ways to treat this parasitic worm infection to gain an optimum chicken health status by following its back-to-nature concept.

Many medicinal plants have antioxidant, antiviral, antibacterial, and antiparasitic activities, including *Phyllanthus niruri* L., *Andrographis paniculata*, *Curcuma xanthorrhiza* Roxb., and *Curcuma aeruginosa* Roxb. The *P. niruri* L. plant has long-standing ethnomedical records from Ayurvedic, Chinese, Malay, and Indonesian. One of the most popular medicinal plants in Asia, America, and Africa is A. paniculata wall (family *Acanthaceae*). The genus *Curcuma* mainly originates from Asia, Australia, and South America, and it has been used for medicinal, aromatic, nutritional, and cosmetic purposes [6].

Our research is a promising hope for the poultry industry. By investigating the anthelmintic properties of *P. niruri* L., *A. paniculata*, *C. xanthorrhiza* Roxb., and *C. aeruginosa* Roxb., we aimed to provide a more affordable and effective treatment for anthelmintic resistance. This study aimed to elaborate on the *in vitro* anthelmintic effects of the ethanol extract of *P. niruri* L., *A. paniculata*, *C. xanthorrhiza* Roxb., and *C. aeruginosa* Roxb. on *A*. *galli* motility, analyze damage to the cuticle, and consider the possibility of using plant extracts as an alternative anthelmintic to parasitic infection.

## Materials and Methods

### Ethical approval

This study was approved by the Animal Laboratory Ethical Committee, School of Veterinary Medicine and Biomedical Sciences, IPB University (Approval No.: 84/KEH/SKE/II/2024).

### Study period and location

The study was conducted from January 2024 to May 2024 in the Veterinary Helminthology Laboratory, Division of Parasitology and Medical Entomology, Division of Veterinary Pathology, Division of Veterinary Pharmacy, School of Veterinary Medicine and Biomedical Sciences IPB University and Tropical Biopharmaca Research Center, IPB University.

### Preparation of the plant’s simplicial

All plants of *P. niruri* L., *A. paniculata*, *C. xanthorrhiza* Roxb., and *C. aeruginosa* Roxb. provided by the Tropical Biopharmaca Research Center at IPB University were washed with tap water and dried. The dried simplicity of all plants was achieved by meshing 20 mesh to obtain the powder form [7].

### Extraction of plants

The extract was prepared according to the book of Indonesian Pharmacopeia, 2^nd^ edition [8]. The dried powder of the plants was extracted using a maceration method for 3 × 24 h with an ethanol concentration of 96%. The ratio of simplicial to ethanol was 1:10. The condensed extracts were obtained by evaporating the extract filtrate using a rotary evaporator at 40°C and 50 rpm and then freeze-dried using a freeze dryer [7].

### Phytochemical analysis

Chemical components were identified using the Vanquish method according to the manufacturer’s instructions (ThermoFisher Scientific, USA). Chemical components of the extracts were examined using a Q Exactive Plus ultra-high-performance liquid chromatography high-resolution mass spectrometer Orbitrap (UHPLC-Q-Orbitrap HRMS, ThermoFisher Scientific) equipped with an Accucore C18 (100 × 2.1 mm, 1.5 μm). The mobile phase used was 0.1% formic acid in water (A) and 0.1% formic acid in acetonitrile (B) with a gradient elution system: 0.0–1.0 min (5% B), 1.0–25.0 min (5%–95% B), 25.0–28.0 min (95% B), and 28.0–33.0 min (5% B). The flow rate was maintained at 0.2 mL/min, with an injection volume of approximately 2 μL. Other parameters for the UHPLC-Q-Orbitrap HRMS analysis were as follows: The source of mass spectrometry ionization was electrospray ionization (+) using a Q-Orbitrap mass analyzer with an m/z range of 100 m/z–1500 m/z. The collision energies used for fragmentation were 18, 35, and 53 eV. The spray voltage was approximately 3.8 kV, the capillary temperature was 320°C, and the sheath and auxiliary gas flow rates were 15 and 3 mL/min, respectively. We used scan-type full MS/dd MS2 for the positive-ion mode. The metabolites were tentatively identified using the acquired mass spectra and analyzed using Compound Discoverer version 3.2. software (https://www.thermofisher.com) in an untargeted metabolomics workflow. Peak extraction was filtered, and the MzCloud (https://www.mzcloud.org) and ChemSpider databases (https://www.chemspider.com) were employed for annotation with mass accuracies between –5 parts per million (ppm) and 5 ppm.

### *In vitro* motility analysis of the adult *A. galli worm*

The samples used in this *in vitro* assay were fresh *A. galli* adult worms under the criteria that the worm was still active in movement [9]. Adult *A. galli* were collected from intestine of the kampung chicken (Indonesian Indigenous chicken/local breed) obtained from the slaughterhouse. The study used a completely randomized design. The worms were placed in a Petri dish. Each Petri dish contained two adult worms for each treatment in three replicates. Each plant extract (*P. niruri* L., *A. paniculata, C. xanthorrhiza* Roxb., and *C. aeruginosa* Roxb.) was evaluated with three distinct doses, which were 250 μg/mL, 500 μg/mL, and 1000 μg/mL [10]. A 0.9% sodium chloride solution was used for the negative control group, and Albendazole 500 μL was used to validate the effectiveness of the plant extracts. The effectivity of worm motility was determined using a scoring activity from 1- to 6-h post-exposure (PE) as follows: score 0: No movement (worm died); score 1: Weak movement; score 2: Moderate movement; and score 3: Active movement.

### Microscopical preparations

The entire *A. galli* specimen was fixed in 10% neutral buffer formalin. The fixated worms were then cut to a thickness of 5 mm from the 1/3 anterior, 1/3 middle, and 1/3 posterior parts of the worm. Tissues were placed in a tissue cassette and then put in the tissue processor for dehydration in graded ethanol followed by the clearing process using a xylol solution. The tissue samples were then embedded in a paraffin block, cut using a rotary microtome with a thickness of ±3–5 μm, and stained with Hematoxylin-Eosin [11]. The microscopical lesion of the worm cuticle was examined using a state-of-the-art Digital MicroscopeVHX-7000 (Keyence, Japan) and an Olympus Photomicroscope BX5 (Olympus, Japan) showcasing the use of advanced equipment in our research.

### Statistical analysis

Statistical analysis was performed using a two-way analysis of variance to determine the effect of plant extracts on *A. galli* from the PE group in 1 h–6 h. Significant differences among treatment groups are indicated by p < 0.05.

## Results

### Phytochemical analysis

**Figure-1:**
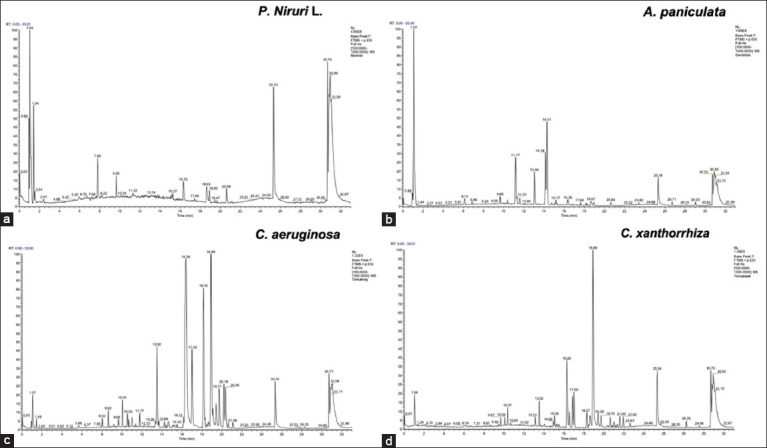
The UPLC-MS chromatogram of (a) *P. niruri* L., (b) *A. paniculata*, (c) *C. aeruginosa* Roxb., and (d) *C. xanthorrhiza* Roxb. Several peaks were detected in all extracts. *P. niruri=Phyllanthus niruri*, *A. paniculata=Andrographis paniculata*, *C. xanthorrhiza* Roxb=*Curcuma xanthorrhiza* Roxb, *C. aeruginosa* Roxb*=Curcuma aeruginosa* Roxb.

Through UPLC-MS analyses, 100 compounds in *P. niruri* L., extracts were identified. The chromatogram diagram is shown in Figure-1a. It contains flavonoids, an alkaloid (phyllanthin), coumarin (linamarin), lignan (5-Methoxybenzimidazole), and phenols (zingerol). In the ethanol extracts of *A. paniculata* (Figure-1b), the detected compounds were terpenoid andrographolide (retention time [RT] 11,265), and 3-O-β-D-glucosyl-14-deoxyandrographolide (RT 14,389 min).

The compound analysis of the *C. aeruginosa* Roxb. ethanol extract (Figure-1c) revealed phenolic curcumin (RT 20,615 min) and terpenoid alantolactone (RT 11,777 min). Similarly, the *C. xanthorrhiza* Roxb. ethanol extract was found to contain phenolic curcumin (RT 16,848 min), curcumin (RT 20,358 min), and terpenoid alantolactone (RT 17,045 min) (Figure-1d). Table-1 shows the phytochemical active compound composition of the ethanol extract.

**Table-1:** Phytochemical active compound composition of the ethanol extract.

No.	Name	Formula	Annot. DeltaMass (PPM)	MW	Retention time (min)	Classification	Plants
1.	(-)-Nupharamine	C_15_H_25_NO_2_	1. 01	251.18878	16.295	Alkaloids	*C. aeruginosa, C. xanthorrhiza*
2.	8-Methyl-8-azabicyclo[3.2.1] oct-3-yl (3S)-1,2- dithiolane-3-carboxylate	C_12_H_19_NO_2_S_2_	−3.5	27.308.476	1.172	Alkaloids	*P. niruri*
3.	1,2,3,4-Tetrahydro-Î²- carboline-3-carboxylic acid	C_12_H_12_N_2_O_2_	00.56	216.09.00	6.416	Alkaloids	*P. niruri*
4.	Phenethylamine	C_8_H_11_N	03.29	12.108.955	0.170833333	Alkaloids	*P. niruri*
5.	Genistein	C_15_H_10_O_5_	0.052083333	27.005.303	12.792	Isoflavone	*A. paniculata*
6.	5-Ethyl-3,8-dimethyl-1, 7-dihydroazulene	C_14_H_18_	1.59	186.14115	17.96	Flavonoids	*C. aeruginosa*
7.	Baicalin	C_21_H_18_O_11_	−0.06	44.608.489	0.426388889	Flavonoids	*A. paniculata*
8.	Luteolin 7-O-glucuronide	C_21_H_18_O_12_	00.39	462.08.00	10.464	Flavonoids	*A. paniculata*
9.	Tectoridin	C_22_H_22_O_11_	00.23	46.211.632	10.083	Flavonoids	*A. paniculata*
10.	Favan-3-ol	C_15_H_14_O_2_	01.09	22.609.981	14.383	Flavonoids	*A. paniculata*
11.	Tangeritin	C_20_H_20_O_7_	00.48	37.212.108	14.567	Flavonoids	*A. paniculata*
12.	Linamarin	C_10_H_17_NO_6_	0.085416667	24.710.599	1.501	Coumarin	*P. niruri*
13.	Meranzin	C_15_H_16_O_4_	00.03	26.010.494	19.511	Coumarin	*P. niruri*
14.	Aesculin	C_15_H_16_O_9_	01.05	34.007.979	6.836	Coumarin	*P. niruri*
15.	Phellopterin	C_17_H_16_O_5_	0.050694444	30.009.999	17.733	Coumarin	*A. paniculata*
16.	4-Hydroxycoumarin	C_9_H_6_O_3_	01.32	16.203.191	6.215	Coumarin	*A. paniculata*
17.	Osthol	C_15_H_16_O_3_	1.36	244.11028	13.129	Coumarin	*C. aeruginosa, C. xanthorrhiza*
18.	5-Methoxybenzimidazole	C_8_H_8_N_2_O	0.100694444	14.806.394	1.157	Lignan	*P. niruri*
19.	Phyllanthin	C_24_H_34_O_6_	0.086111111	41.823.622	0.8	Lignan	*P. niruri*
20.	Zingerol	C_11_H_16_O_3_	0.063888889	19.611.012	9.178	Phenol	*P. niruri*
21.	Curcumin	C_21_H_20_O_6_	0.76	368.12627	16.848	Phenol	*C. aeruginosa, C. xanthorrhiza*
22.	Curcumin II	C_20_H_18_O_5_	1.57	338.11595	16.544	Phenol	*C. aeruginosa, C. xanthorrhiza*
23.	Thymol	C_10_H_14_O	2.02	150.10477	21.35	Phenol	*C. xanthorrhiza*
24.	Damascenone	C_13_H_18_O	00.53	19.013.587	17.323	Volatile oil	*P. niruri*
25.	Dehydrocostus lactone	C_15_H_18_O_2_	00.52	2.301.308	0.815972222	Terpenes	*P. niruri*
26.	(-)-Andrographolide	C_20_H_30_O_5_	−0.75	35.020.906	11.265	Terpenes	*A. paniculata*
27.	3-O- β-D-glucosyl-14- deoxyandrographolide	C_26_H_40_O_9_	−0.19	49.626.714	11.41	Terpenes	*A. paniculata*
28.	Linalyl benzoate	C_17_H_22_O_2_	−0.14	25.816.194	14.242	Terpenes	*A. paniculata*
29.	Valerenic acid	C_15_H_22_O_2_	−0.42	234.16188	16.384	Terpenes	*A. paniculata*
30.	(+)-Alantolactone	C_15_H_20_O_2_	1.2	232.14661	18.586	Terpenes	*C. aeruginosa, C. xanthorrhiza*
31.	(±)-(2E)-Abscisic acid	C_15_H_20_O_4_	0.45	264.13628	11.376	Terpenes	*C. aeruginosa, C. xanthorrhiza*
32.	Helenalin	C_15_H_18_O_4_	0.55	262.12065	13.138	Terpenes	*C.aeruginosa*
33.	Curcumene	C_15_H_22_	0.61	202.17227	20.615	Terpenes	*C. aeruginosa, C. xanthorrhiza*
34.	(+)-Nootkatone	C_15_H_22_O	0.79	218.16724	21.595	Terpenes	*C. xanthorrhiza*
35.	(E, E)-alpha-Farnesene	C_15_H_24_	1.53	204.18811	22.613	Terpenes	*C. xanthorrhiza*
36.	Pristimerin	C_30_H_40_O_4_	1.29	464.29326	26.153	Terpenes	*C. xanthorrhiza*
37.	3,4-Dihydrocadalene	C_15_H_20_	0.97	200.15669	21.594	Terpenes	*C. xanthorrhiza*

*P. niruri=Phyllanthus niruri, A. paniculata=Andrographis paniculata, C. xanthorrhiza=Curcuma xanthorrhiza,* *C. aeruginosa=Curcuma aeruginosa*, PPM=Parts per million, MW=Molecular weight

### *In vitro* motility analysis of the adult *A. galli worm*

**Figure-2:**
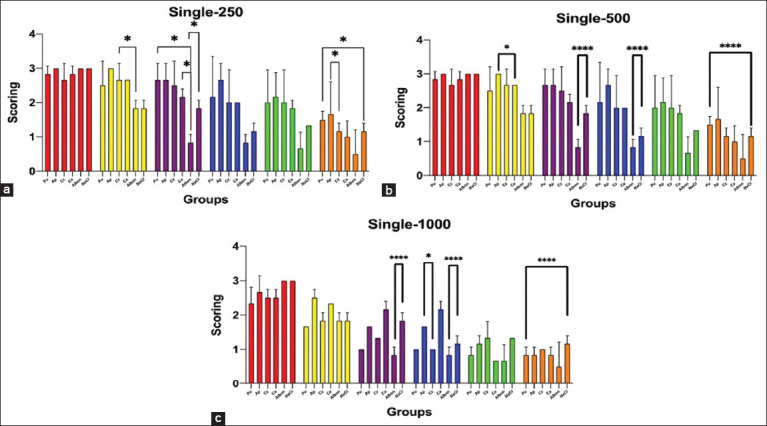
*In vitro* assay of the ethanol extract of *A. paniculata, P. niruri* L., *C. xanthorrhiza* Roxb., and *C. aeruginosa* Roxb. on *A. galli* motility in several single doses of the extracts. (a) Exposure to a single dose of 250 μg/mL, (b) Exposure to a single dose of 500 μg/mL, and (c) Exposure to a single dose of 1000 μg/mL. Pu: *P. niruri* L.; AP: *A. paniculta*; Cz: *C xanthorrhiza* Roxb; Ca: *C. aeruginosa* Roxb; Alben: Albendazole; sodium chloride (NaCl): 0.9% NaCl solution. The differences in the bar color groups indicate the different times of PE: Red: 1 h; Yellow: 2 h; Brown: 3 h; Blue: 4 h; Green: 5 h; and Orange: 6 h. The asterisk symbol indicates significant differences among the extracts within the same hour of observation (p < 0.05 = *, p < 0.01 = **, p < 0.001 = ***, p < 0.0001 = ***). *A. galli*=*Ascaridia galli*, *P. niruri=Phyllanthus niruri*, *A. paniculata=Andrographis paniculata*, *C. xanthorrhiza* Roxb=*Curcuma xanthorrhiza* Roxb, *C. aeruginosa* Roxb*=Curcuma aeruginosa* Roxb.

Treatment of *A. galli* with the ethanol extracts of *A. paniculata, P. niruri* L., *C. xanthorrhiza* Roxb., and *C. aeruginosa* Roxb. in a single dose of 250 μg/mL, 500 μg/mL, and 1000 μg/mL revealed a different response on the motility score of *A. galli* from 1 to 6 h PE (Figure-2). In the 1-h PE in all tested doses of all plant extracts, including the positive and control groups, the effect of antimotility on *A. galli* was undetected, and all worms were still active and alive (Figure-2). In the 2-h PE, the response was mainly similar to that in the 1-h exposure, except at doses of 250 μg/mL and 500 μg/mL for *C. xanthorrhiza* Roxb. and *A. paniculata*, there was a significant difference (p < 0.05) (Figure-2a and b).

Our study found that the 250 μg/mL dose during the 1-h PE of *P. niruri* L., *C. aeruginosa* Roxb., *A. paniculata*, and *C. xanthorrhiza* Roxb. did not affect the *A. galli* motility (Figure-2a). However, we observed a significant decrease in *A. galli* motility (p < 0.05) in control, *P. niruri* L., and *C. xanthorrhiza* Roxb. groups at 2-h PE. At 3 h PE, we noted significant differences (p < 0.05) in the decreasing motility of *A. galli* in the positive control group compared with the negative control group and the groups of *C. aeruginosa* Roxb. and *P. niruri* L. There were no differences in the motility of *A. galli* in the herbal treatment groups compared with the control groups at 4 and 5 h of PE. Notably, significant differences (p < 0.05) were detected in the groups of *P. niruri* L. with the group of negative control and the group of *A. paniculata* with the group of *C. xanthorrhiza* Roxb. at 6 h of PE.

At a dose of 500 μg/mL in 1-h PE of all extract plants and control groups, similar results were obtained as with a dose of 250 μg/mL, where there was no inhibition of motility of *A. galli* (Figure-2b). At 2-h PE, there were no differences in the motility inhibition of *A. galli* in all extract plants and the control groups, except for *A. paniculata* and *C. aeruginosa* Roxb. (p < 0.05). Significant differences (p < 0.001) existed between the positive and negative control groups in the 3- and 4-h PE. At 5 h of PE, there were no differences in the inhibition motility of *A. galli* between the extract and control groups. Significant differences (p < 0.001) in decreasing motility of *A. galli* at the 6 h PE in the *P. niruri* L. and negative control groups.

In the 1000 μg/mL dose, all plant extracts significantly influenced the motility of *A. galli* (Figure-2c). At 1 h of PE, a decrease in *A. galli* motility was observed compared with the control group, although the difference was not significant. However, after 2 h of PE, *A. galli* motility was decreased compared with that after 1 h of PE in all extracts and control groups. At 3-h PE, the *A. galli motility* weakened further than that at 2 h PE, with highly significant differences (p < 0.001) in the positive and negative control groups. At 4-h PE, *A. galli* motility was significantly reduced in the *P. niruri* L. *and C. xanthorrhiza* Roxb. groups, with a significant difference (p < 0.05) between *A. paniculata* and *C. xanthorrhiza* Roxb. The motility of *A. galli remained* similar to that of 4 and 5 h of PE. At 6 h of PE, the motility of *A. galli* was significantly reduced in all plant extract groups and the positive and negative control groups, with significant differences (p < 0.0001) in *A. galli* motility in the negative control and *P. niruri* L. groups.

### Microscopical observation

**Figure-3:**
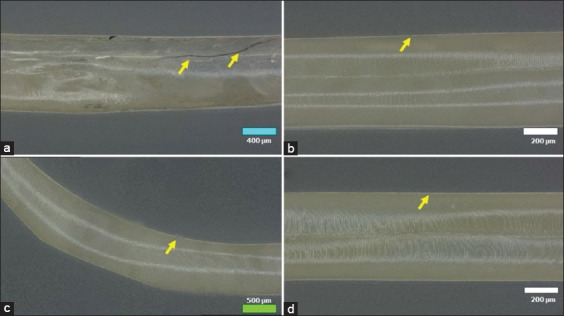
Photomicroscope using a nano microscope of *A. galli* cuticle exposed to the plant extracts with 1000 μg/mL dose during the 6-h PE. (a) Cracking cuticle layer (arrow) in the *A. paniculata* extract group, (b) A thin layer of *A. galli* cuticle (arrow) in the *C. xanthorrhiza* Roxb. extract group, (c) Cuticle with some desquamated and erosion (arrow) in the *P. niruri* L. extract group, and (d) A thin layer of *A. galli* cuticle (arrow) in the *C. aeruginosa* Roxb. extract group. Bar = 100 μm. *A. galli*=*Ascaridia galli*, *P. niruri=Phyllanthus niruri*, *A. paniculata=Andrographis paniculata*, *C. xanthorrhiza* Roxb=*Curcuma xanthorrhiza* Roxb, *C. aeruginosa* Roxb*=Curcuma aeruginosa* Roxb, PE=Post-exposure.

**Figure-4:**
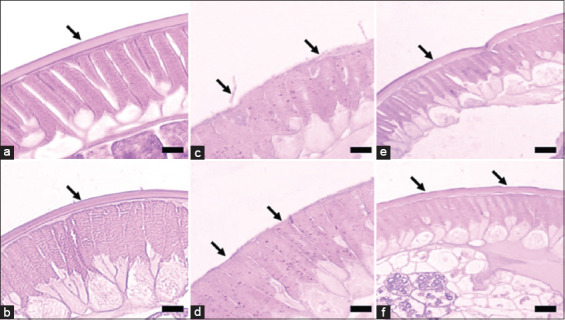
Histopathological picture of the *A. galli* cuticle exposed to 1000 μg/mL of plant extracts at 6 h PE. (a) Normal cuticle from the negative control group; (b) Cuticle from the positive control group, similar to the negative control group; (c) Cuticle exposed to *A. paniculata* extract, the cuticle was desquamated, erosion, and thin with irregular surface (arrow); (d) Cuticle exposed to *P. niruri* L. extract, similar to the *A. paniculata* extract group (arrow). (e) Cuticle exposed to *C. xanthorrhiza* Roxb. extract, the lesions of the cuticle were thin, moderately desquamated in some parts, and irregularity of the cuticle surface (arrow); F: Cuticle exposed to *C. aeruginosa* Roxb. extract, the lesions were similar to the *C. xanthorrhiza* Roxb. extract group. Hematoxylin-Eosin staining. *A. galli*=*Ascaridia galli*, *P. niruri=Phyllanthus niruri*, *A. paniculata=Andrographis paniculata*, *C. xanthorrhiza* Roxb=*Curcuma xanthorrhiza* Roxb, *C. aeruginosa* Roxb*=Curcuma aeruginosa* Roxb, PE=Post-exposure.

Microscopical lesions in the *A. galli* cuticles were detected in all plant extract groups, and all tested doses started from 2 h to 6 h PE with a gradual gradation of the lesions from the thin cuticle, irregularity of the surface, cracking cuticle wall, erosion, and desquamation of the cuticle layer. Severe lesions in the cuticle were primarily detected in 1000 μg/mL of all plant extract at the 6-h PE. The 1000 μg/mL *A. paniculata* and *P. niruri* L. extracts caused the most severe lesions in the *A. galli* cuticle layer. Observation using a nano-microscope revealed the cracking part of the cuticle wall caused by exposure to the 1000 μg/mL *A. paniculata* extract at 6 h PE, as shown in Figure-3a. The same severe lesion with desquamation and erosion of the cuticle layer of *A. galli* was detected on observation by histopathology assay in *A*. galli exposed to 1000 μg/mL *A. paniculata* and *P. niruri* L. extracts, as shown in (Figures-3a and c, 4c and d). On the other hand, there were moderate lesions of irregularity and depletion of the cuticle of *A. galli* following exposure to the extracts of *C. xanthorrhiza* Roxb. and *C. aeruginosa* Roxb. (Figures-3b and 3d, 4e, and f). Importantly, no lesions were found in the cuticle layer of the *A. galli* in the positive and negative control groups (Figures-4a and b).

## Discussion

Ascaridiasis, a prevalent and noteworthy poultry disease, is caused by the soil-transmitted helminth *A. galli* (Schrank, 1788), which is the largest nematode in chickens and the most frequently encountered problem in indigenous chickens [12]. *A. galli* infection involves a thickened intestinal wall with petechial hemorrhage, edema, and infiltration of lymphoid cells mixed with eosinophils [13]. The intestinal epithelium acts as a communication network for this gut-dwelling nematode; thus, gastrointestinal nematode infection could cause damage to the mucosal epithelial cells of the chicken’s digestive tract and increased mucus production, leading to desquamation, adhesion of mucous villi, epithelial cell necrosis, and goblet cell hyperplasia [14, 15]. *A. galli* infection not only results in problems with nutrient absorption but also has complex immunomodulatory effects that can alter the host’s immune response to the disease [16].

The results of this study revealed the potential of herbal plant extracts as treatments for *A. galli*. It was found that increasing the single extract dose and observation time significantly affected *A. galli* motility. The active compounds from *P. niruri* L. extract, such as flavonoids, alkaloids (phyllanthin), coumarin (linamarin), lignans (5-Methoxybenzimidazole), and phenolics (zingerol), appear to cause *A. galli* cuticles to desquamate and erosion, resulting in the cuticle becoming thin with an irregular surface, which influences the weakening of *A. galli* motility. It is worth noting that Oxfendazole, an anthelmintic derivative of Benzimidazole [17], remains a highly effective treatment for *A. galli* [18], providing reassurance in the fight against this parasite. Furthermore, the metabolite of *A*. *paniculata* containing the terpenoid Andrographolide can damage and cause desquamation and erosion of the cuticle layer, resulting in an irregular surface and decreased *A. galli* motility. The results of another study stated that Thymoquinone, the main terpenoid compound in black cumin seed (*Nigella sativa*), has an anthelmintic strong activity in reducing the motility of *A. galli* and damages the tegument of *Paramphistomum* spp. [19, 20]. Further, the phenolic curcumin and terpenoid alantolactone metabolite compounds from *C. xanthorrhiza* Roxb. and *C. aeruginosa* Roxb. extracts caused the cuticle to become thin, slightly desquamate, and irregular, inhibiting *A. galli* motility. Another study by Mubarokah *et al*. [21] reported that tannin and saponin in *Areca catechu* crude aqueous extract cause morphological changes in adult *A. galli*.

The role of secondary metabolites of herbal plants with anthelmintic activity, such as terpenes (glycosides and saponins), phenolics (alkaloids and tannins), and nitrogen content (alkaloids, cyanogenic glycosides, and non-protein amino acids), is fascinating due to their diverse mechanisms. These mechanisms include damaging the worm’s mucopolysaccharide membrane, which affects the worm’s active movement, inhibiting the worm’s fecundity, and damaging the worm’s cuticle [22]. It is understandable that the cuticle is the main target for deworming. The nematode cuticle, a complex extracellular structure, is metabolically active and morphologically varies between the genera of worms, larvae, and adults. It consists of three parts, with many layers containing glycoproteins and lipids. However, specific collagen and insoluble protein (cuticlin) truly define the nematode cuticle. These two components, which are rich in the cuticle, are crucial in structure and function. The epicuticle, which forms the cuticle’s outermost layer, also contains insoluble proteins. The middle layer of the cuticle is the matrix divided into a fibril layer containing aromatic amino acids and a thick homogeneous layer consisting of albumin protein and fibrous proteins resembling fibrin or elastin, as well as carbohydrates, lipids, and esterase enzymes [23].

Previous studies [4, 5, 21, 24, 25] have been conducted on the effects of medicinal plants on poultry worms. Clove leaf extract (*Syzygium aromaticum*) was reported to change the surface and damage the cuticle of *A. galli*, resulting in the death of *A. galli* at 3, 6, and 9 h after exposure to 140 mg/mL of the clove leaf ethanol extract [26]. At a dose of 100 mg/mL, the ethanol extract of *Juglans regia* L. leaves inhibited the motility of adult *A. galli* by 96.5% 24 h after exposure [5]. The ethanol extract of black cumin seeds at a concentration of 45% can kill *A. galli* in 10 h [20]. The water extract of *Areca catechu* can cause morphological changes and result in the death of *A. galli* [21]. *Nyctanthes arbor-tristis* and *Butea monosperma* leaf extracts have significant *in vivo* anthelmintic activity against *A. galli* [24]. *Mimosa pudica* leaf ethanol extract and *Carica papaya* seed extract were also reported to reduce egg per gram feces and affect blood and fat parameters in Kabir chickens infected with *A. galli* in Cameroon [25]. Anthelmintic effects were observed at a 20% *Jatropha curcas* Linn leaf extract concentration against *A. galli* [27]. The crude methanol extract of *Saussurea costus* inhibits worm motility inhibition in *A. galli* at 100 mg/mL after 24 h of exposure [4]. In addition to anthelmintic activity against gastrointestinal parasitic infections, anti-protozoan activity against gastrointestinal protozoan parasitic infections has been reported. Curcumin, a polyphenol from turmeric (*Curcuma* spp.), has been known to have anti-coccidial effects [28]. Curcumin is also known to have anti-malarial activity [29].

Various worm medicines such as Albendazole, Piperazine, Levamisole, and Ivermectin are often used to control *A. galli*. The use of anthelmintics for an extended period can result in worm resistance. Some researchers have proven that fenbendazole is resistant to *Ascaridia dissimilis*, a digestive tract worm in turkey [30]. Since a vaccine for digestive tract worms, especially *A. galli* has not yet been developed, the worm control program has been based only on administering worm medicine. This study proposes a control approach for the ethanol extracts of *A*. paniculata, *P. niruri* L., C. *xanthorrhiza* Roxb., and *C. aeruginosa* Roxb. as medicinal plants with anthelmintic activity against *A. gall*i. The use of herbal plant-based anthelmintics in chickens can significantly impact parasitology by reducing the development of anthelmintic resistance. Furthermore, the need for *in vivo* studies to assess the efficacy of *A. paniculata*, *P. niruri* L., *C. xanthorrhiza* Roxb., and *C. aeruginosa* Roxb. as anthelmintics in chickens infected with *A. galli* is paramount. These studies will provide valuable insights into the effects of these medicinal plants in natural infection, enhancing our understanding of their potential as anthelmintics. This research is necessary and interesting as it will serve as a reference for future *in vivo* studies using chickens and guide us toward more effective worm disease control strategies.

## Conclusion

The ethanol extracts of *A. paniculata, P. niruri* L., *C. xanthorrhiza* Roxb., and *C. aeruginosa* Roxb. have shown promising anthelmintic activity at a concentration of 1000 μg/mL. Notably, the extracts of *A. paniculata* and *P. niruri* L. at this concentration exhibit intense anthelmintic activity, damaging and dissolving the cuticle layer of *A. galli*, thereby weakening the ability of adult *A. galli* to move. These findings hold great potential for the development of novel anthelmintic treatments.
